# Socioeconomic Status is Not Associated With Complications Following Revision Total Joint Arthroplasty

**DOI:** 10.1155/aort/1303184

**Published:** 2025-12-03

**Authors:** Reece I. Vesperman, Vincent Young, Alicia M. Hymel, Anoop S. Chandrashekar, Emily R. Oleisky, J. Ryan Martin

**Affiliations:** ^1^ Department of Orthopaedic Surgery, Vanderbilt University Medical Center, Nashville, Tennessee, USA, vanderbilt.edu; ^2^ Department of Orthopaedic Surgery, Vanderbilt University School of Medicine, Nashville, Tennessee, USA, vanderbilt.edu

**Keywords:** Distressed Communities Index, revision, socioeconomic status, total hip arthroplasty, total knee arthroplasty

## Abstract

Socioeconomic disparities are known to affect outcomes after primary total hip and knee arthroplasty. Revision procedures may be similarly associated, though this relationship remains unclear. This study examined the association between socioeconomic status (SES), measured by the Distressed Communities Index (DCI), and short‐term complication rates following revision total hip (rTHA) and knee arthroplasty (rTKA).This retrospective cohort study included 851 consecutive revision arthroplasties (502 rTKAs, 349 rTHAs) performed between January 1st, 2018, and January 1st, 2025, with at least 6 months of follow‐up. Preoperative and postoperative data were collected via chart review. Multivariate regression analyses were conducted, adjusting for demographic and clinical variables. Alpha was set at 0.05. Higher Charleston Comorbidity Index (CCI) was associated with increased 90‐day medical complications in both rTHA and rTKA (*p* < 0.001). Higher EBL was also associated with greater medical complications for rTHA patients (*p* = 0.048). Younger age, higher CCI, and greater operative time correlated with higher surgical complication rates in rTKA (*p* < 0.05). DCI was associated with increased postoperative opioid use in rTHA patients and decreased 30‐day readmission in rTKA patients (*p* = 0.005 and 0.025, respectively). However, after adjusting for pain, DCI was not independently associated with 90‐day complications. *Post hoc* analysis demonstrated adequate statistical power. SES, as measured by the DCI, was not independently associated with short‐term postoperative complications following rTHA or rTKA.

**Clinical Significance:**

These findings suggest that DCI and similar indices used in isolation may have limited utility in risk‐stratifying revision arthroplasty patients.

## 1. Introduction

Revision total joint arthroplasty (rTJA) procedures are increasingly more common due to the aging population and expanding indications for primary TJA (pTJA) [1]. Historically, revision hip and knee arthroplasties have been associated with higher complication rates and greater healthcare costs compared to primary arthroplasty [2, 3]. Despite advances in surgical techniques, modern implant designs, and contemporary imaging modalities, outcomes for revision procedures continue to lag behind those of primary arthroplasty. Revision procedures remain burdened by higher rates of prosthetic joint infections (PJIs), worse functional outcomes, and increased hospital and healthcare system utilization [4, 5].

In response, recent emphasis has been placed on optimizing outcomes for rTJA by identifying and understanding patient‐level risk factors. Prior studies have demonstrated associations between several risk factors and poor outcomes following pTJA, including socioeconomic status (SES) [6–9]. However, the impact of SES on rTJA outcomes remains poorly understood. Existing literature surrounding pTJA and social determinants of health may not be generalizable to revision populations, as patients undergoing rTJA are often older, more medically complex, and face the inherent risks of repeat surgery not seen in their pTJA counterparts [10, 11]. Therefore, studies must be tailored to this population to meaningfully inform clinical practice.

The Distressed Communities Index (DCI) is a composite measure developed by the Economic Innovation Group that incorporates seven economic and social indicators of SES using data from the U.S. Census Bureau [12]. The DCI stratifies zip codes into quintiles ranging from “prosperous” to “distressed.” DCI was chosen over other SES measures because it integrates both static (poverty rate and educational attainment) and dynamic (job growth) indicators into a single standardized distress score. While previously employed in the pTJA literature [9], the utility of the DCI in predicting complication rates after revision arthroplasty remains untested. If shown to be associated with rTJA complications, DCI could serve as a valuable tool to guide surgical decision‐making, inform referral practices, and optimize resource allocation.

The primary objective of our study was to determine whether the DCI is associated with 90‐day medical or surgical complications following rTJA. Secondarily, we aimed to identify other demographic and clinical predictors of 90‐day complications. Finally, we assessed the relationship between DCI and healthcare utilization, including opioid use and hospital readmissions. We hypothesized that higher DCI scores, reflecting more distressed communities and lower SES, would be associated with greater medical and surgical complication rates and increased healthcare utilization.

## 2. Materials and Methods

### 2.1. Study Population

This was a single‐center retrospective cohort study conducted at a tertiary academic institution. The study was approved by the institutional review board (IRB) prior to data collection. Patients were initially identified via our institutional clinical data repository. We included all patients older than 18 years of age who were identified as having undergone either a revision total hip (rTHA) or revision total knee arthroplasty (rTKA) by a board‐certified, fellowship‐trained, orthopedic surgeon. A minimum 6‐month postoperative follow‐up period was required. Exclusion criteria included patients aged < 18 years, missing demographic or outcome data, or insufficient follow‐up. Ultimately, we identified 851 patients who underwent rTJA between January 1st, 2018, and January 1st, 2025.

Data collected included patient demographics (age, sex, and race), risk factors (body mass index, smoking status, and medical comorbidities), preoperative pain scores, and postoperative outcome metrics (pain scores, readmission rate, surgical complications, and medical complications). Medical comorbidities were assessed by calculating the Charlson Comorbidity Index (CCI) and the American Society of Anesthesiologists Physical Status (ASA). Demographic details for the patient population are reported in Tables 1 and 2. Primary outcomes of interest were medical and nonpain‐related surgical complications within 90 days of rTJA. Secondary outcomes of interest were pain‐related outcomes including readmission rates associated with pain, severe postoperative pain (defined as pain uncontrolled by the current analgesic regimen), and opioid requirements.

**Table 1:** Summary of patient characteristics.

Characteristic	Hip = 349, *n* (%)	Knee = 502, *n* (%)
Sex		
Male	163 (46.7)	240 (47.8)
Female	186 (53.3)	262 (52.2)
Age (years)		
Mean (SD)	60.9 (13.6)	64.0 (10.2)
Median [IQR]	62.0 [53.0, 70.0]	65.0 [58.0, 71.0]
CCI		
Mean (SD)	4.13 (3.2)	4.4 (2.9)
Median [IQR]	3.0 [2.0, 6.0]	4.0 [2.0, 6.0]
ASA score		
1.0	1 (0.3)	4 (0.80%)
2.0	87 (24.9)	125 (24.9)
3.0	254 (72.8)	361 (71.9)
4.0	7 (2.0)	12 (2.4)
Weight		
Normal weight	68 (19.5)	45 (8.9)
Underweight	5 (1.4)	6 (1.2)
Overweight	107 (30.7)	125 (24.9)
Obese	144 (41.3)	244 (48.6)
Morbidly obese	25 (7.2)	82 (16.3)
Pre‐op BMI		
Mean (SD)	30.3 (6.6)	32.9 (6.9)
Median [IQR]	29.5 [25.7, 34.9]	32.50 [28.3, 37.7]
Smoking status		
Current	41 (16.9)	41 (11.3)
Former	79 (32.6)	123 (33.8)
Never	122 (50.4)	200 (55.0)
Unknown	107.0	138.0
Baseline pain score (0–10)		
Mean (SD)	2.8 (3.13)	2.9 (3.2)
Median [IQR]	2.0[0.0, 5.0]	2.00 [0.0, 5.0]
Operative time		
Mean (SD)	160.7 (70.7)	168.2 (64.9)
Median [IQR]	141.0 [113.0, 201.0]	161.5 [122.3, 203.0]
DCI score (higher = more distressed)		
Mean (SD)	49.8 (29.2)	48.4 (29.5)
Median [IQR]	49.4 [25.5, 74.4]	47.6 [20.2, 75.3]
DCI quintile		
1 Prosperous	78 (22.4)	123 (24.5)
2 Comfortable	42 (12.0)	78 (15.5)
3 Mid‐tier	78 (22.4)	98 (19.5)
4 At risk	89 (25.5)	101 (20.1)
5 Distressed	62 (17.8)	102 (20.3)
DCI (3‐category)		
Least disadvantaged 20%	78 (22.4)	123 (24.5)
Middle 60%	209 (59.9)	277 (55.2)
Most disadvantaged 20%	62 (17.8)	102 (20.3)
Estimated blood loss (mL)		
Mean (SD)	348.3 (336.4)	227.1 (242.4)
Median [IQR]	250.0 [150.0, 450.0]	150.0 [100.0, 300.0]

Abbreviations: BMI = body mass index; CCI = Charleston Comorbidity Index; DCI = Distressed Communities Index; IQR = interquartile range; SD = standard deviation.

**Table 2:** Summary of patient characteristics based on DCI score.

Characteristic	Hip, *N* = 349			Knee, *N* = 502		
	Least disadvantaged 20%, *N* = 78	Middle 60%, *N* = 209	Most disadvantaged 20%, *N* = 62	Least disadvantaged 20%, *N* = 123	Middle 60%, *N* = 277	Most disadvantaged 20%, *N* = 102
Sex						
Male	40 (51.28%)	90 (43.06%)	33 (53.23%)	64 (52.03%)	131 (47.29%)	45 (44.12%)
Female	38 (48.72%)	119 (56.94%)	29 (46.77%)	59 (47.97%)	146 (52.71%)	57 (55.88%)
Age						
Mean (SD)	64.33 (14.22)	60.21 (13.39)	59.05 (13.12)	64.98 (10.34)	64.19 (10.32)	62.20 (9.51)
Median [IQR]	67.00 [58.25, 74.00]	62.00 [52.00, 70.00]	61.00 [50.00, 67.75]	65.00 [59.50, 71.00]	65.00 [58.00, 72.00]	62.00 [56.00, 70.00]
CCI						
Mean (SD)	4.51 (3.21)	4.22 (3.20)	3.35 (2.89)	4.47 (3.06)	4.41 (2.72)	4.41 (3.08)
Median [IQR]	4.00 [2.25, 6.00]	4.00 [2.00, 6.00]	2.00 [2.00, 4.00]	4.00 [2.50, 6.00]	4.00 [2.00, 6.00]	4.00 [2.00, 6.00]
ASA score						
1	0 (0.00%)	1 (0.48%)	0 (0.00%)	0 (0.00%)	4 (1.44%)	0 (0.00%)
2	26 (33.33%)	48 (22.97%)	13 (20.97%)	38 (30.89%)	65 (23.47%)	22 (21.57%)
3	52 (66.67%)	154 (73.68%)	48 (77.42%)	85 (69.11%)	200 (72.20%)	76 (74.51%)
4	0 (0.00%)	6 (2.87%)	1 (1.61%)	0 (0.00%)	8 (2.89%)	4 (3.92%)
Weight (categorical)						
Normal weight	16 (20.51%)	40 (19.14%)	12 (19.35%)	12 (9.76%)	26 (9.39%)	7 (6.86%)
Underweight	0 (0.00%)	5 (2.39%)	0 (0.00%)	2 (1.63%)	2 (0.72%)	2 (1.96%)
Overweight	32 (41.03%)	60 (28.71%)	15 (24.19%)	31 (25.20%)	78 (28.16%)	16 (15.69%)
Obese	27 (34.62%)	85 (40.67%)	32 (51.61%)	58 (47.15%)	132 (47.65%)	54 (52.94%)
Morbidly obese	3 (3.85%)	19 (9.09%)	3 (4.84%)	20 (16.26%)	39 (14.08%)	23 (22.55%)
Pre‐op BMI						
Mean (SD)	29.16 (5.50)	30.62 (7.18)	30.75 (6.08)	32.09 (6.75)	32.74 (6.48)	34.67 (7.29)
Median [IQR]	28.50 [25.68, 32.27]	29.90 [25.70, 35.40]	30.90 [25.75, 35.48]	32.30 [27.50, 35.70]	32.10 [27.80, 37.30]	34.45 [30.00, 39.60]
Smoking status						
Current	6 (11.11%)	24 (17.14%)	11 (22.92%)	4 (4.55%)	22 (11.28%)	15 (18.52%)
Former	18 (33.33%)	46 (32.86%)	15 (31.25%)	24 (27.27%)	72 (36.92%)	27 (33.33%)
Never	30 (55.56%)	70 (50.00%)	22 (45.83%)	60 (68.18%)	101 (51.79%)	39 (48.15%)
Unknown	24	69	14	35	82	21
Baseline pain score (0–10)						
Mean (SD)	1.54 (2.59)	3.08 (3.16)	3.18 (3.28)	2.99 (3.24)	2.83 (3.19)	2.92 (3.34)
Median [IQR]	0.00 [0.00, 2.00]	3.00 [0.00, 6.00]	3.00 [0.00, 6.00]	2.00 [0.00, 5.50]	2.00 [0.00, 5.00]	1.50 [0.00, 6.00]
Operative time (minutes)						
Mean (SD)	144.77 (52.27)	158.46 (69.48)	188.42 (86.54)	151.64 (60.06)	175.82 (67.81)	167.55 (58.78)
Median [IQR]	135.00 [112.00, 182.00]	139.00 [110.00, 194.00]	176.50 [120.75, 227.75]	146.00 [113.00, 176.50]	171.00 [128.00, 210.00]	162.00 [126.25, 199.25]
DCI score (higher = more distressed)						
Mean (SD)	7.08 (5.89)	54.40 (16.11)	88.05 (5.31)	9.28 (6.34)	51.38 (17.38)	87.28 (4.70)
Median [IQR]	5.30 [1.64, 10.08]	51.27 [44.59, 69.06]	87.78 [83.00, 92.12]	8.57 [4.03, 15.07]	49.25 [39.63, 67.21]	86.47 [82.98, 90.97]
DCI quintile						
1 Prosperous	78 (100.00%)	0 (0.00%)	0 (0.00%)	123 (100.00%)	0 (0.00%)	0 (0.00%)
2 Comfortable	0 (0.00%)	42 (20.10%)	0 (0.00%)	0 (0.00%)	78 (28.16%)	0 (0.00%)
3 Mid‐tier	0 (0.00%)	78 (37.32%)	0 (0.00%)	0 (0.00%)	98 (35.38%)	0 (0.00%)
4 At risk	0 (0.00%)	89 (42.58%)	0 (0.00%)	0 (0.00%)	101 (36.46%)	0 (0.00%)
5 Distressed	0 (0.00%)	0 (0.00%)	62 (100.00%)	0 (0.00%)	0 (0.00%)	102 (100.00%)
Estimated blood loss (mL)						
Mean (SD)	259.62 (170.16)	363.90 (372.62)	407.18 (347.95)	211.40 (297.92)	230.57 (193.20)	236.75 (285.96)
Median [IQR]	200.00 [150.00, 350.00]	250.00 [150.00, 450.00]	300.00 [160.00, 500.00]	150.00 [50.00, 200.00]	200.00 [100.00, 300.00]	200.00 [100.00, 300.00]

Abbreviations: BMI = body mass index; CCI = Charleston Comorbidity Index; DCI = Distressed Communities Index; IQR = interquartile range; SD = standard deviation.

### 2.2. DCI

To obtain individual patient DCI scores, patient zip code information was utilized to identify the appropriate score from the DCI database. This database is publicly available for a fee (https://eig.org/distressed-communities-index/). Patients were then categorized by DCI scores into most disadvantaged (top 20% of scores), middle (middle 60% of scores), and least disadvantaged (bottom 20% of scores) groups.

### 2.3. Data Analyses

Statistical analysis was performed to determine the association between DCI scores and postoperative complication rates. Categorical variables were analyzed using Pearson’s chi‐square tests, and continuous variables were analyzed using the Wilcoxon rank–sum test. Logistic regression was conducted to adjust for potential confounders. Subgroup analyses were performed separately for rTKA and rTHA patients. Both septic and aseptic revisions were included. Patients undergoing revision for a PJI were excluded from the “PJI” surgical complication list to more accurately reflect the true postrevision PJI incidence in our dataset. Medical and surgical complications were categorized based on the anatomic region in which the complication occurred. Surgical complications were defined as those related to the operative hip or knee. Medical complications involved organs outside of the hip or knee. Pain outcomes were analyzed separately. Opioid use was quantified based on the milligrams of morphine equivalents (MMEs) given to the patient via IV, while inpatient and orally at discharge. Pain management protocols utilized multimodal regimens but were not standardized and therefore inconsistent across the study period. Alpha was set at 0.05 for significance. Finally, a post hoc analysis was performed to ensure an adequate sample size. All data analyses were performed using R Studio 2024 (RStudio PBC, Boston, Massachusetts, USA).

## 3. Results

### 3.1. SES and Cohort Characteristics

**Figure 1:**
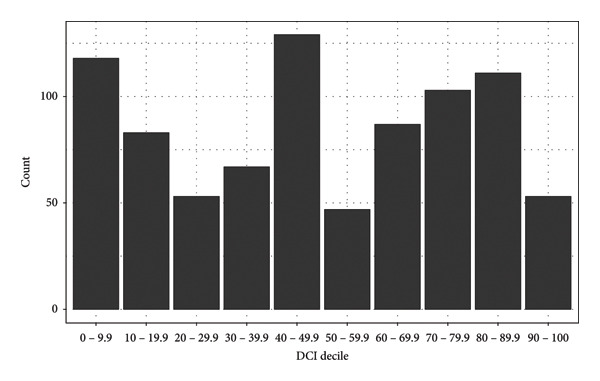
Distribution of the Distressed Communities Index in the patient population.

In total, 851 patients were identified meeting inclusion and exclusion criteria including 502 rTKAs (59%) and 349 rTHAs (41%). When utilizing DCI to stratify patients, the distribution followed a multimodal curve, with a median score of 49.4 (interquartile range [IQR]: [25.5–74.4]) and 47.1 (IQR: [20.2–75.3]) for rTHA and rTKA, respectively (Figure 1). Most patients were within the middle 60% of the DCI distribution, including 60% of rTHA patients and 55% of rTKA patients (Tables 1 and 2). The rTKA cohort had a higher percentage of patients within the distressed quintile (20%) and prosperous quintile (25%) when compared to the rTHA cohort (18% and 22%, respectively). The median age was 62 years for rTHA and 65 years for rTKA (IQR: [53–70] and [58–71], respectively). Across all rTHA, 47% patients were men and 48% of rTKA patients were men. The median CCI score was 3 for rTHA and 4 for rTKA (IQR: [2–6]). A majority of patients had an average ASA score of 3 for both rTHA (*n* = 254, 73%) and rTKA (*n* = 361, 72%). Likewise, the majority of patients were classified as obese in both the rTHA (*n* = 144, 42%) and rTKA (*n* = 244, 49%) groups. The median preoperative BMI for rTHA patients was 30 kg/m^2^ (SD: 6.66) and 33 kg/m^2^ for rTKA patients (IQR: [25.7–34.9] and [28.8–37.7], respectively). At least half of all patients were never‐smokers at the time of rTJA (rTHA = 50.4% and rTKA = 55.0%). Median baseline pain scores (graded 0–10) were 2 for both groups (IQR: [0.0–5.0]). Operative time showed a median of 141 min for rTHA (IQR: [113–201]) and 161.5 min for rTKA (IQR:[122.3–203]). Estimated blood loss (EBL) was higher amongst rTHA patients with a median of 250 mL (IQR: [150–450]) compared to 150 mL for rTKA patients (IQR: [100–300]).

### 3.2. Ninety‐Day Complications

A total of 52 (14.9%) rTHA and 66 (13%) rTKA patients suffered at least one medical complication within 90 days of surgery, the most common being pulmonary edema (Table 3). Excluding postoperative pain, 108 (31%) rTHA and 47 (9%) rTKA patients suffered at least one surgical complication within 90 days of surgery (Table 4). The most common nonpain–related surgical complication was dislocation for rTHA and PJI for rTKA (15% and 4%, respectively, Table 4). Analysis of both cohorts showed no significant difference (*p* > 0.05) for 90‐day medical complications when comparing DCI quintiles (Table 5). Likewise, when excluding pain, multilevel analysis showed DCI was nonsignificant (*p* > 0.05) in predicting surgical complications for both rTJA cohorts (Table 6). However, subgroup analysis with severe pain included as a postoperative outcome showed that a lower DCI score was associated with higher rates of 90‐day surgical complications for rTKA (*p* = 0.048, Table 7).

**Table 3:** 90‐day medical complications in the cohort.

Characteristic	Hip = 349, *n* (%)	Knee = 502, *n* (%)
Any medical complications	52 (14.9)	66 (13.2)
MI	3 (0.9)	1 (0.2)
CVA	1 (0.3)	4 (0.8)
PE/DVT	13 (3.7)	19 (3.8)
Acute respiratory failure	7 (2.0)	7 (1.4)
Pulmonary edema	28 (8.0)	27 (5.4)
Pneumonia	3 (0.9)	5 (1.0)
Sepsis	9 (2.6)	16 (3.2)
Urinary tract infection	8 (2.3)	10 (1.9)

Abbreviations: CVA = cerebrovascular accident; DVT = deep vein thrombosis; MI = myocardial infarction; PE = pulmonary embolism.

**Table 4:** 90‐Day surgical complications in the cohort.

Characteristic	Hip = 349, *n* (%)	Knee = 502, *n* (%)
Any surgical complications	198 (56.7)	314 (62.6)
Any surgical complications (excluding pain)	108 (30.9)	47 (9.4)
Mechanical loosening	25 (7.2)	18 (3.6)
Dislocation	52 (14.9)	3 (0.6)
PJI	20 (5.7)	19 (3.8)
PPFx	22 (6.3)	1 (0.2)
Pain	147 (42.1)	295 (58.8)
Hematoma or wound complication	16 (4.6)	12 (2.4)

*Note:* Pain, pain uncontrolled by the current analgesic regimen (inpatient and at follow‐up); PPFx = periprosthetic fracture.

Abbreviation: PJI = prosthetic joint infection.

**Table 5:** Regression analysis of medical complications based on predictors.

Predictors	Hip revisions, *n* = 349			Knee revisions, *n* = 502		
	Odds ratios	CI	*p*	Odds ratios	CI	*p*
DCI score (higher = more distressed)	1.0	0.989–1.013	0.9	1.0	0.988–1.007	0.5
Age	1.0	0.964–1.024	0.6	1.0	0.997–1.068	0.1
Sex: female	1.2	0.607–2.341	0.6	1.0	0.583–1.768	1.0
CCI	1.3	1.180–1.473	**< 0.001**	1.2	1.082–1.304	**< 0.001**
Pre‐op BMI	1.0	0.923–1.027	0.4	1.0	0.988–1.076	0.2
Baseline pain score (0–10)	1.1	0.973–1.193	0.2	1.0	0.923–1.094	0.9
Operative time	1.0	0.996–1.006	0.7	1.0	0.999–1.008	0.2
Estimated blood loss	1.0	1.000–1.002	**0.0**	1.0	0.999–1.001	0.6

*Note:* Bold = *p* < 0.05; P, alpha.

Abbreviations: CI = confidence interval; DCI = Distressed Communities Index.

**Table 6:** Regression analysis of surgical complications based on predictors.

Predictors	Hip revisions, *n* = 349			Knee revisions. *n* = 502		
	Odds ratios	CI	*p*	Odds ratios	CI	*p*
DCI score (pain excluded)	1.0	0.991–1.008	0.9	1.0	0.792–1.187	0.8
Age	1.0	0.961–1.001	0.1	1.0	0.932–0.978	**< 0.001**
Sex: female	1.2	0.784–1.930	0.4	1.4	0.923–1.982	0.1
CCI	1.1	0.997–1.192	0.1	1.1	1.053–1.248	**0.003**
Pre‐op BMI	1.0	0.968–1.035	1.0	1.0	0.978–1.038	0.6
Baseline pain score (0–10)	1.0	0.953–1.101	0.5	1.0	0.982–1.110	0.2
Operative time	1.0	0.995–1.004	0.8	1.0	1.002–1.009	**0.004**
Estimated blood loss	1.0	1.000–1.002	0.1	1.0	0.999–1.001	0.9

*Note:* Bold = *p* < 0.05; DCI = Distressed Communities Index (higher = more distressed).

Abbreviations: BMI = body mass index; CCI = Charleston Comorbidity Index; CI = confidence interval.

**Table 7:** Pain‐related outcomes and opioid usage stratified by DCI score.

Predictors	Hip revisions, *n* = 349										Knee revisions, *n* = 502									
	30‐day readmission (including pain‐related)		Post‐op IV opioids		Discharge MMEs		Surgical complications (pain included)		LOS		30‐day readmission (including pain‐related)		Post‐op IV opioids		Discharge MMEs		Surgical complications (pain included)		LOS	
	OR (CI)	*p*	OR (CI)	*p*	Beta (CI)	*p*	OR (CI)	*p*	OR (CI)	*p*	OR (CI)	*p*	OR (CI)	*p*	Beta (CI)	*p*	OR (CI)	*p*	OR (CI)	*p*
DCI score (higher = more distressed)	1.00 (0.99–1.02)	0.848	1.01 (1.00–1.02)	**0.005**	0.17 (0.05–0.29)	**0.005**	0.998 (0.990–1.006)	0.993	0.00 (−0.01–0.01)	0.763	0.98 (0.97–1.00)	**0.025**	1.01 (1.00–1.01)	0.08	0.02 (−0.08–0.12)	0.692	0.993 (0.987–1.00)	**0.048**	0.00 (−0.00–0.01)	0.592

*Note:* Bold = *p* < 0.05; IV, intravenous; LOS, hospital length of stay.

Abbreviations: CI = confidence interval; DCI = Distressed Communities Index; MMEs = milligrams of morphine equivalents; OR = odds ratio.

Higher CCI scores were significantly associated with 90‐day medical complications for both rTHA and rTKA (*p* < 0.001, Table 5). In addition, higher EBL was associated with greater medical complication rates in rTHA patients (*p* = 0.048, Table 5). Similarly, higher CCI was significant in predicting 90‐day surgical complications for rTKA (*p* = 0.003, Table 6). For rTKA patients, longer operative time was significantly associated with 90‐day surgical complications (*p* = 0.004, Table 6). Younger age was predictive of surgical complications in rTKA (*p* < 0.001, Table 6). rTHA patients with higher DCI scores required significantly more IV opioid, while inpatient, and were discharged on larger quantities of opioid medications (*p* = 0.005, Table 7). DCI score was not associated with postoperative hospital length of stay for revision hips or knees (Table 7). Finally, rTKA with higher DCI scores displayed lower 30‐day hospital readmission rates when pain control was included as a reason for admission (Table 7).

## 4. Discussion

Social, cultural, and economic factors have previously been shown to influence outcomes following TJA [9, 13, 14]. Composite metrics such as the DCI have been developed to efficiently stratify patients based on SES using zip code–level data [12]. In our study of rTJA patients, DCI was not independently associated with 90‐day‐medical or surgical complications after adjusting for relevant covariates. However, DCI was inversely related to 90‐day surgical complications in our rTKA cohort when severe pain was evaluated as a postoperative outcome. In other words, less distressed or more prosperous patients were more likely to report uncontrolled pain within 90 days of their rTKA. This finding is in line with literature showing lower patient satisfaction and functional outcomes when TKA is compared to THA [15, 16]. Furthermore, more prosperous patients may have higher expectations, while less prosperous patients may have financial constraints, limiting follow‐up adherence and leading to underreporting of symptoms such as poorly controlled pain in the acute postoperative period. These findings contribute to an evolving and somewhat mixed body of literature on the relationship between neighborhood‐level SES and arthroplasty complication rates.

A recent study by Chandrashekar et al. examined over 4000 pTJA patients using DCI and similarly found that while DCI correlated with demographic and health‐related risk factors, it did not predict short‐term complications, reoperations, or revision surgeries at 90‐day [9]. Though potentially underpowered when compared to pTJA literature, our results parallel their findings, reinforcing the notion that DCI may capture social context but may not independently predict short‐term clinical outcomes after arthroplasty, particularly once clinical confounders are controlled.

Conversely, Bains et al., using the Area Deprivation Index (ADI), did find that higher neighborhood socioeconomic disadvantage was independently associated with increased 90‐day complications following pTJA [14]. Their results suggest that more granular, census block–level SES measures such as ADI may capture risk in a way that broader ZIP code–based tools such as DCI do not. However, it is worth noting that Bains et al. focused exclusively on pTJA, whereas our study addresses the rTJA population, a group that is generally older, more medically complex, and at uniformly higher surgical risk. These baseline differences may attenuate the relative impact of SES in the revision setting.

We also observed mixed results when evaluating SES in the context of healthcare utilization. Higher DCI scores were associated with increased inpatient opioid use and greater quantities of opioid prescriptions at discharge following rTHA. Conversely, higher DCI scores were also associated with lower readmission rates following rTKA. However, DCI was also not predictive of medical comorbidities or reoperation rates. These findings suggest that while SES may be associated with certain patterns of perioperative resource use, it may not consistently translate to increased medical or surgical complications in the revision context.

In line with prior research in the primary arthroplasty literature, we found that higher CCI score, increased EBL, and longer operating time were strong, independent predictors of postoperative complications [17–19]. Interestingly, we also noted that younger patients in our cohort experienced more surgical complications, possibly due to more complex or atypical indications for undergoing revision TJA at an earlier age.

Our study has several limitations. Its retrospective, single‐center design may limit generalizability and may not be applicable to all regions or hospital systems. Although DCI is validated, zip code–based indices alone may not fully capture individual‐level social determinants of health. Zip codes can be relatively large and heterogenous with wealthy and poor neighborhoods coexisting in the same zip code. Therefore, extremes of poverty or wealth may be obscured and average out when using this metric. Moreover, our analysis was limited to 90‐day outcomes; longer‐term follow‐up could reveal more delayed SES‐related disparities. In impoverished areas with tenuous job stability and healthcare coverage, long‐term outcomes after rTJA may be more revealing than the initial 3‐month post‐op period. In addition, postoperative pain management was not standardized secondary to different admitting teams and patient‐specific comorbidities, which may confound our ability to interpret pain‐related outcomes. Nonetheless, our work is among the first to investigate DCI in the revision arthroplasty population, offering a nuanced contribution to a literature that is still largely centered on primary procedures. Future studies should explore a combination of SES indices across multiple centers and include longer follow‐up to account for regional variation and other social risk factors. In addition, future revision arthroplasty risk models may include SES as measured by DCI to mitigate confounding variables.

## 5. Conclusion

The DCI score was not an independent predictor of 90‐day medical or surgical complications following revision TJA. While SES is undoubtedly an important factor in shaping patient outcomes, these findings suggest that DCI and similar zip code–based indices, when used in isolation, may have limited utility for risk stratification at the individual level in this high‐risk, medically complex revision population. A more nuanced and granular approach to measuring SES, potentially incorporating individual‐level or neighborhood block‐level data, may be necessary to more accurately assess risk and guide clinical decision‐making in revision arthroplasty care.

## Disclosure

All authors listed have read and approved the final submitted manuscript.

## Conflicts of Interest

The authors declare no conflicts of interest.

## Author Contributions

Reece I. Vesperman, Vincent Young, Alicia M. Hymel, Anoop S. Chandrashekar, and Emily R. Oleisky assisted with the research design, data acquisition and analysis, and interpretation of data, as well as drafting of the paper. J. Ryan Martin, Reece I. Vesperman, and Emily R. Oleisky assisted in revising and critically analyzing the paper. Approval of final versions of the paper was performed by Reece I. Vesperman, J. Ryan Martin, and Alicia M. Hymel.

## Funding

No funding was received for this study.

## Data Availability

The data that support the findings of this study are available from the corresponding author upon reasonable request.
